# The Medical Addresses

**Published:** 1899-10-07

**Authors:** 


### The Hospital

A Journal of

The Medical Sciences and Hospital Administration.

Vol. XXVII.—No. 680. Edited by Sir Henry Burdett, K.C.B., and Solomon C. Smith, M.D., M.R.C.P. [October 7, 1899.

### The Medical Addresses.

The 1st, or rather, this year, the 2nd of October, has brought forth its usual abundance of addresses at the medical schools of London and of the provinces; the institutions at which this time-honoured custom has been abandoned being still in a very decided, we will not say insignificant, minority. The provincial schools have especially distinguished themselves by bringing eminent talent from afar; so that Sir James Crichton Browne has been speaking at Manchester, and Professor Sir William Gairdner at Birmingham. In London, if we may name any one of the orators as *primus inter pares*, we should be inclined to assign that distinction to Dr. Dickinson, whose address at St. George's Hospital is described by those who heard it as concrete of wisdom, illustrated by epigrams, and as containing the fruits of long and ripe experience condensed into sentences so constructed as to linger in the memory. Most unfortunately, the abstract prepared for communication to the lay Press, although it could not be entirely divested of the wisdom, afforded no adequate presentment of the epigrams; and hence we trust that the address will, in some form or other, be ultimately published as it was delivered. One of Dr. Dickinson's strongest points had reference to the practical inexpediency of treating such a symptom as elevation of temperature by the routine administration of the so-called "antipyretic" medicines by which temperature may be reduced; and his warning abundantly illustrated the not uncommon belief that, during the epidemic of influenza which prevailed a few years ago, many young physicians, who rapidly reduced the abnormal temperature of their patients by such means, were almost inviting the occurrence of the pneumonia by which, before long, the cases were hurried to a fatal issue. Dr. Dickinson expressed his belief that high temperature was frequently of the nature of a combustion by which noxious matters were "burnt and purged away," and that any interference with this process tended rather to the retention of a *materies morbi* than to its elimination. He mentioned one case in his own experience in which the temperature had reached 111 deg., and in which the patient had recovered without the employment of a single dose of any antipyretic remedy; and, even if it be conceded that a person of sceptical disposition might not unfairly ask to have the thermometer employed in the observation once more tested at Kew, it is at least impossible to doubt that the case was one in which an elevation commonly thought to be incompatible with recovery was far exceeded. The lesson was an eminently practical one, and it fitted with peculiar propriety into an address of which the main subject was the gradual passage of medicine from empiricism into science.

Sir James Crichton Browne is always prone to adorn the least attractive subjects with suggestions of eminent originality, and to derive from the dryest of dry bones suggestions replete with fancy and adorned by eloquence. He spoke at Birmingham on "The Quest of the Ideal," and dwelt chiefly on the necessity of not placing the imagination under the fetters of materialism. He claimed that the greatest thinkers of the medical profession had always avoided materialism, and had reconciled faith with knowledge; and quoted Sir John Lubbock as having said that the four great men of science whom he had helped to carry to their last resting place in Westminster Abbey—Lyell, Herschell, Spottiswoode, and Darwin, had all done something to promote the cause of true religion. He might have added to his examples the name of one, perhaps more illustrious still—the name of Faraday, who constantly declared that he held his scientific and his religious faiths upon totally different bases, and that, while regarding the former as a proper subject for examination, for inquiry, and for modification by reasoning and by experiment, he regarded the latter as something standing apart, into which the light of reason could not penetrate, and which had to be received and accepted on grounds practically independent of evidence. Sir James described materialism, naturalism, and agnosticism as three formidable giants which are always lying in wait to devour medical students; and he urged them to cultivate idealism as the best defence, or, as he calls it, the sure refuge, against the attacks of these insidious enemies.

It is at least certain that the problems of the world are more than puzzling; and that the recorded experience of mankind cannot be held to afford any important assistance in the work of resolving them. The question returns, "Are they soluble?" Even if they are, does not their solution await a period of development more advanced than that which the human race has so far reached; and, while we may wait hopefully for the future, will it not be our wisest course to abandon fruitless speculations, and to direct our efforts towards the attainable? Certain things are within our reach, and it is our plain duty to endeavour to secure them. It is in no sense our duty to neglect the work ready to our hands, in order to perplex our minds over the unknowable.

